# Population genetics and molecular xenomonitoring of *Biomphalaria* freshwater snails along the southern shoreline of Lake Malawi, Malawi

**DOI:** 10.1186/s13071-024-06546-5

**Published:** 2024-12-18

**Authors:** John Archer, Lucas J. Cunningham, Alexandra Juhász, Sam Jones, Amber L. Reed, Shi Min Yeo, Bright Mainga, Priscilla Chammudzi, Donales R. Kapira, David Lally, Gladys Namacha, Peter Makaula, James E. LaCourse, Sekeleghe A. Kayuni, Bonnie L. Webster, Janelisa Musaya, J. Russell Stothard

**Affiliations:** 1 Department of Tropical Disease Biology, Liverpool School of Tropical Medicine, Liverpool, L3 5QA UK; 2 Wolfson Wellcome Biomedical Laboratories, Department of Zoology, Natural History Museum, Cromwell Road, London, UK; 3 Institute of Medical Microbiology, Semmelweis University, Budapest, 1089 Hungary; 4 Department of Clinical Research, London School of Hygiene and Tropical Medicine, Keppel Street, London, UK; 5 Laboratory Department, Mangochi District Hospital, P.O. Box 42, Mangochi, Malawi; 6 Malawi-Liverpool-Wellcome Trust Clinical Research Programme, Queen Elizabeth Central Hospital, Blantyre, Malawi; 7 Department of Pathology, School of Medicine and Oral Health, Kamuzu University of Health Sciences (KUHeS), Blantyre, Malawi

**Keywords:** *Biomphalaria pfeifferi*, *Schistosoma mansoni*, Intestinal schistosomiasis, Malawi, Phylogenetics, Molecular xenomonitoring, Water sanitation and hygiene (WASH), Waterborne pathogens, One Health

## Abstract

**Background:**

Intestinal schistosomiasis was confirmed endemic in Mangochi District, Malawi, in May of 2018 following an unexpected encounter with discreet populations of *Biomphalaria* spp. freshwater snails during routine malacological surveillance activities. Since then, only limited malacological surveillance of *Biomphalaria* has been carried out, and so the distribution of *Biomphalaria* populations in this area is currently unclear. Additionally, sites of active *Schistosoma mansoni* transmission in this area are also unknown. In the present study, through extensive malacological surveillance, we aimed to formally document the distribution of *Biomphalaria* in Mangochi District. We also aimed to identify active intestinal schistosomiasis transmission sites in this area through subjecting all collected *Biomphalaria* to a recently developed *S. mansoni-*specific molecular xenomonitoring PCR.

**Methods:**

Three malacological surveys were carried out along the southern shoreline of Lake Malawi, Mangochi District, Malawi, in November 2021, July 2022 and October/November 2022. All collected *Biomphalaria* were subjected to cercarial shedding analysis to identify active *Schistosoma* infections. Shed cercariae were then genotyped to species level using a standard multi-locus PCR and Sanger sequencing protocol. Following this, a subset of *Biomphalaria* from each collection site were also genotyped to species level using a standard PCR and Sanger sequencing protocol. All collected *Biomphalaria* were then subjected to a recently developed *S. mansoni-*specific molecular xenomonitoring PCR to identify infected, but non-shedding, *Biomphalaria.*

**Results:**

A total of 589 *Biomphalaria* were collected across all three surveys. One single *Biomphalaria* (0.17%) specimen was found to be actively shedding *Schistosoma* cercariae, which were molecularly confirmed as *S. mansoni*. All genotyped *Biomphalaria* (*n* = 42) were molecularly identified as *B. pfeifferi*. A further 19 *Biomphalaria* specimens, collected from four different surveillance sites, were found to be infected with *S. mansoni* through molecular xenomonitoring. Intestinal schistosomiasis transmission was therefore identified at four different foci in Mangochi District.

**Conclusions:**

Our study highlights the importance of molecular approaches to investigate *Biomphalaria* populations and monitor *Biomphalaria*-associated intestinal schistosomiasis transmission in endemic areas. As such, the continued development and use of such approaches, in particular the development and use of molecular xenomonitoring assays that can be carried out in resource-poor schistosomiasis-endemic settings, is encouraged. The revision of ongoing schistosomiasis control programmes in Mangochi District, in line with WHO recommendations, is also encouraged.

**Graphical abstract:**

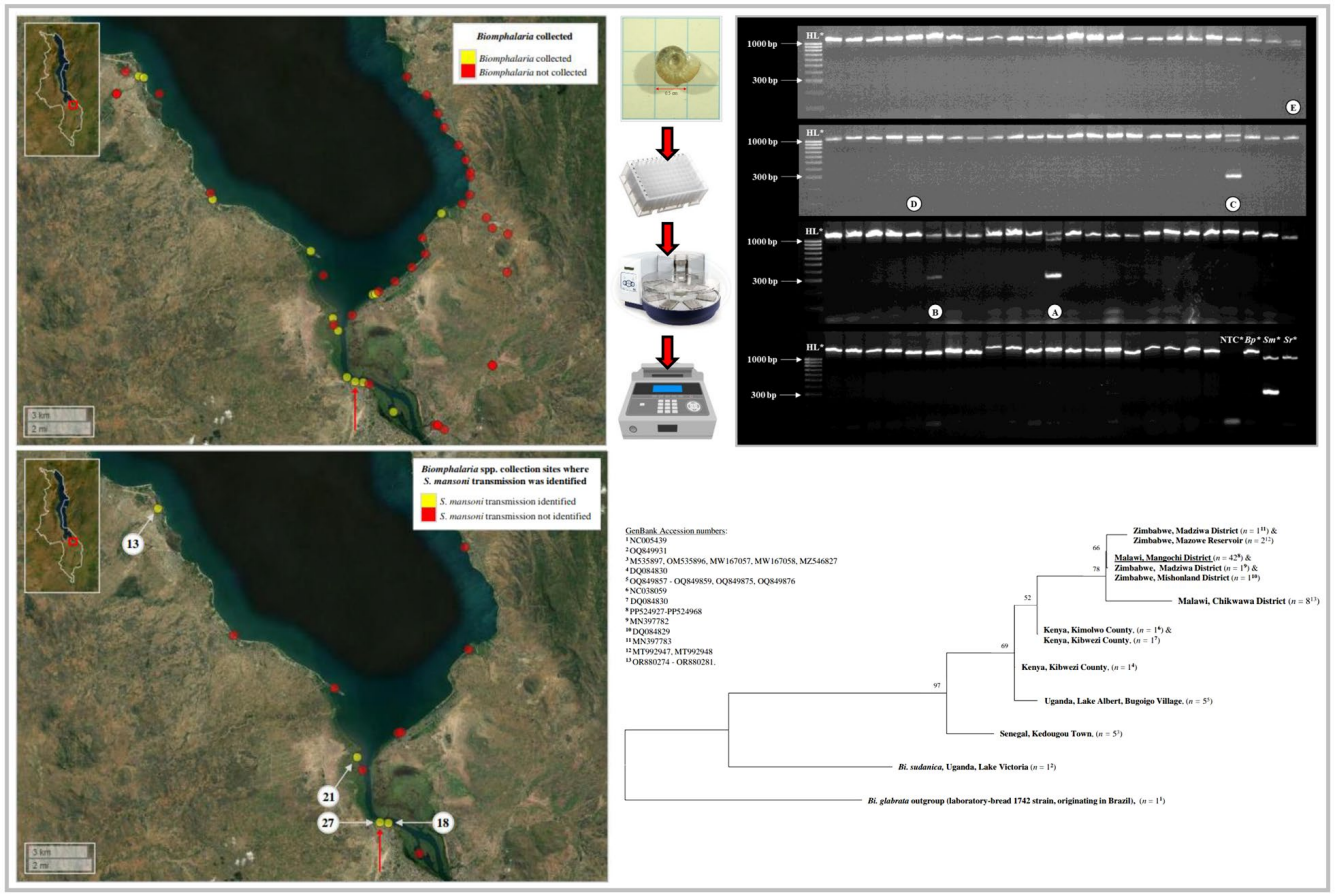

**Supplementary Information:**

The online version contains supplementary material available at 10.1186/s13071-024-06546-5.

## Background

Schistosomiasis, caused by parasitic trematodes of the genus *Schistosoma*, is a neglected tropical disease (NTD) infecting over 230 million people worldwide [1]. Infection with these parasites can lead to debilitating morbidity and mortality. More than 90% of schistosomiasis cases occur within sub-Saharan Africa [2], and approximately one-third of these cases are deemed intestinal schistosomiasis, caused predominantly by infection with *Schistosoma mansoni* but also less commonly by infection with *Schistosoma intercalatum* and *Schistosoma guineensis* in some restricted areas of central Africa [3, 4]. Human infection occurs through contact with bodies of freshwater contaminated with *S. mansoni* cercariae that have been shed from obligate freshwater snail intermediate hosts of the genus *Biomphalaria* (Gastropoda: Planorbidae) [5]. Intestinal schistosomiasis is therefore often highly prevalent in rural areas lacking adequate water, sanitation and hygiene (WASH) infrastructure [6].

As each *Biomphalaria* species can differ in its ability to transmit *S. mansoni* [7, 8], malacological surveillance and accurate species identification of *Biomphalaria* in intestinal schistosomiasis-endemic areas is not only of taxonomic interest but can also contribute to a better understanding of disease transmission dynamics and risk of human infection. However, differentiating between *Biomphalaria* species based on morphological features, such as shell conchology, is difficult and requires specially trained and skilled malacologists [9]. To overcome these challenges, molecular methods such as PCR are routinely used to reliably identify and differentiate *Biomphalaria* species, typically though genotyping of mitochondrial cytochrome oxidase subunit 1 (*cox*1) DNA, mitochondrial 16S DNA and/or the nuclear internal transcribed spacer (ITS) DNA region [10].

Malacological surveillance together with the collection of *Biomphalaria* can also allow sites of active intestinal schistosomiasis transmission to be identified through cercarial shedding analysis [8, 11, 12]. However, this approach can be misleading owing to morphologically indistinguishable human-infecting and non-human-infecting trematode cercariae, such as *S. mansoni* and *Schistosoma rodhaini*, both of which are transmitted by *Biomphalaria* [13, 14]. As such, molecular methods are also needed to reliably identify the species of* Schistosoma* cercariae shed from *Biomphalaria* hosts. This is typically done through genotyping both mitochondrial (maternally inherited) and nuclear (inherited equally from both parents) DNA loci, as this multi-locus approach allows any potential *Schistosoma* spp. hybrids with recent multi-species ancestry to be identified [15, 16]. In addition, cercarial shedding can also be insensitive, primarily because not all freshwater snails harbouring mature, or patent, *Schistosoma* infections will actively shed cercariae at collection and because freshwater snails harbouring prepatent infections will also not shed cercariae [8, 11, 12]. For these reasons, the World Health Organization (WHO) recommends molecular xenomonitoring of the freshwater snail intermediate hosts of *Schistosoma*, particularly in low-endemicity areas, as this approach can be used to detect *Biomphalaria* infections with *S. mansoni* missed by cercarial shedding [13, 17, 18].

Lake Malawi is one of seven African Great Lakes and has an approximate water surface area of 29,600 km^2^. The lake’s most southern shoreline boarders Mangochi District, Malawi. Here, because WASH infrastructure is inadequate, many people rely on the lake as a source of drinking water and food (via fishing) and as a place to bathe, to tend livestock and for recreation. As such, human water-contact with the lake’s shoreline is commonplace, meaning waterborne diseases in this area, such as schistosomiasis and giardiasis, are highly prevalent [19–21]. Until recently, intestinal schistosomiasis was not considered to be locally endemic in Mangochi District as no species of *Biomphalaria* was known to inhabit the southernmost shoreline of Lake Malawi. However, during routine malacological surveillance in November of 2017, discreet populations of *B. pfeifferi* snails were unexpectedly encountered in submerged beds of *Vallisneria* freshwater vegetation [22]. Subsequently, in May of 2018, an outbreak of intestinal schistosomiasis was confirmed despite annual and ongoing mass drug administration (MDA) campaigns in this area used to reduce transmission of urogenital schistosomiasis [20].

One year later (in 2018), malacological surveillance and cercarial shedding of *Biomphalaria* was carried out in an attempt to identify intestinal schistosomiasis transmission sites in this area [22]. Despite this effort, only one of 201 collected *Biomphalaria* snails was found to be actively shedding *S. mansoni* cercariae. As such, a subset of collected *Biomphalaria* (*n* = 76) snails were screened for *S. mansoni* infection using a real-time molecular xenomonitoring PCR targeting the genus-specific *Schistosoma* ITS2 region. Whilst *Schistosoma* DNA was detected in a high proportion of the screened *Biomphalaria* (31%), it is unclear whether this was derived from invading *S. mansoni* or *Schistosoma rodhaini* trematodes, or even potentially from *S. haematobium-*group trematodes that had penetrated the soft tissues of *Biomphalaria* snails but failed to establish an infection. It is also possible that other species of non-*Schistosoma* trematodes may cross-react with this ITS2-targeting primer/probe combination [23]. Consequently, more extensive molecular xenomonitoring of *Biomphalaria* collected in this area, using a *S. mansoni*-specific assay, is needed to fully assess the prevalence of *Biomphalaria* infections with *S. mansoni* and identify intestinal schistosomiasis transmission sites.

In the present study, through extensive malacological surveillance, we aimed to formally document the distribution of *Biomphalaria* along the southern shoreline of Lake Malawi. We also aimed to identify active intestinal schistosomiasis transmission foci in this area through subjecting all collected *Biomphalaria* to a recently developed *S. mansoni-*specific molecular xenomonitoring PCR [24]. The purpose of this study was therefore to gain a more thorough understanding of *Biomphalaria*-associated transmission of intestinal schistosomiasis in Mangochi District 5 years post-outbreak to better inform and strengthen future disease control efforts.

## Methods

**Fig. 1:**
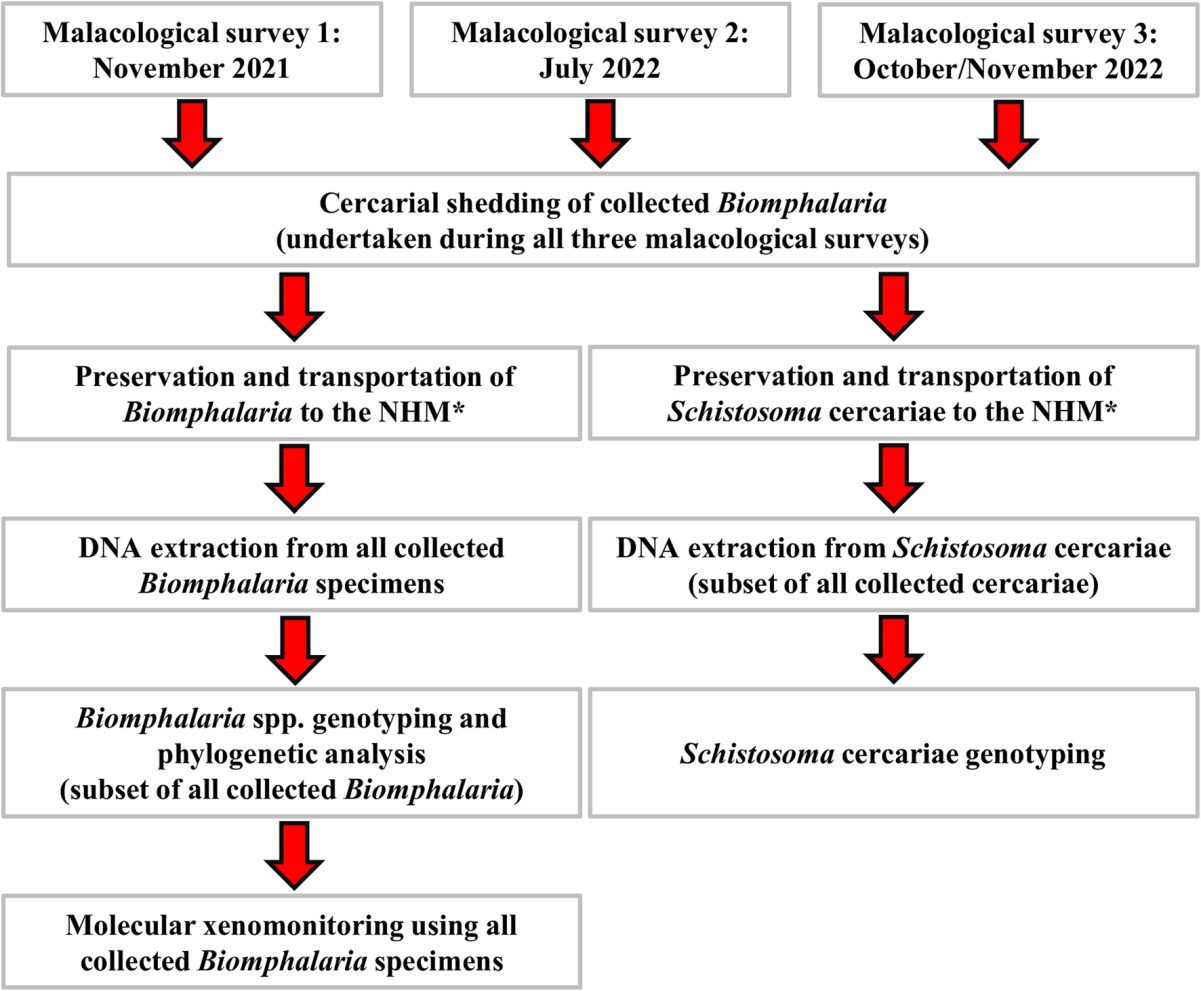
Schematic flow-diagram outlining study design. NHM, Natural History Museum, London, UK

A schematic flow-diagram outlining the study design is shown in Fig. 1.

### Malacological surveys and sample sites

Three malacological surveys were carried out along, and near to, the southern shoreline of Lake Malawi, Mangochi District, Malawi on November 2021 (survey one), July 2022 (survey two) and October/November 2022 (survey three). All surveys were carried out in conjunction with human parasitological surveillance activities, which themselves were timed relative to ongoing MDA with praziquantel in the area. Both purposeful and opportunistic sampling took place; some sampling sites were selected as they are known human-water contact sites and/or *Biomphalaria* had been found at these sites previously, and some sampling sites were selected during surveillance activities as human-water contact was observed and/or because environmental conditions appeared favourable for the presence of *Biomphalaria.* In total, 41 individual sites were surveyed at least once during all three surveys (see Additional file 1: Table S1).

### Collection of* Biomphalaria* freshwater snails

Freshwater snails were collected by scooping using handheld metal sieve scoops according to a standard protocol [18]. Collections were carried out by three trained and experienced technicians for 15 min per sampling site. Brief descriptions of all sampling sites (e.g. water depth, degree of vegetation, whether sites were temporal pools located away from the lake’s edge or were at the lake’s edge) were recorded. The same sampling protocol was applied regardless of the environmental conditions at the sample site. *Biomphalaria* were initially identified to genus level based on shell morphology according to previous morphological descriptions [25] and collected into small screwcap pots containing lake water and lake vegetation for short-term (< 3 h) transportation. Shortly after collection, *Biomphalaria* were maintained in aquaria, also containing lake water and lake vegetation, according to collection site.

### Cercarial shedding of *Biomphalaria* and collection of *Schistosoma* cercariae

Cercarial shedding was carried out according to a standard protocol [26]. In brief, the day following collection, *Biomphalaria* were transferred from aquaria to small transparent plastic ‘shedding’ pots according to collection site. Each shedding pot contained approximately 100 ml of clean, bottled water and no more than 30 *Biomphalaria* specimens. Shedding pots were then placed in a shaded outdoor area (and so not in direct sunlight) between 9:00 a.m. and 4:00 p.m. to coincide with *S. mansoni* cercarial shedding patterns, as described previously [27]. Following light exposure, the water was decanted into a clean petri dish and examined under a dissecting microscope to check for motile, furcocercous *Schistosoma* cercariae according to previous morphological descriptions [28]. Any groups of *Biomphalaria* found to be shedding *Schistosoma* cercariae were recorded, and all *Biomphalaria* were then returned to their original aquaria.

The day following initial cercarial shedding, any groups of *Biomphalaria* snails found to be shedding *Schistosoma* cercariae were then individually separated into wells of a 12-well ‘shedding’ plate containing approximately 5 ml of clean, bottled water. As described above, shedding plates were then also placed in the same shaded outdoor area between 9:00 a.m. and 4:00 p.m. and then examined under a dissecting microscope for the presence of *Schistosoma* cercariae. Any individual *Biomphalaria* found to be shedding *Schistosoma* cercariae was recorded and its corresponding cercariae were individually captured using a micropipette (2.5 µl) and deposited onto Whatman FTA cards for DNA preservation (Whatman, GE Healthcare, Little Marlow, UK). A description of which *Biomphalaria* snail had shed which preserved cercariae was then written onto the FTA cards, and the FTA cards were then stored at ambient temperature according to manufacturer’s instructions.

A record of all shedding and non-shedding *Biomphalaria* was made, and any shedding *Biomphalaria* were individually preserved in 100% ethanol within a labelled 2 ml screwcap tube whereas any non-shedding *Biomphalaria* were pooled according to collection site and preserved in 100% ethanol in labelled 20 ml glass screwcap tubes. All FTA cards and ethanol-preserved *Biomphalaria* were transported to the Natural History Museum (NHM) under ambient conditions for molecular analyses.

### Molecular characterisation of *Biomphalaria*

#### DNA extraction: ethanol-preserved *Biomphalaria*

DNA was individually isolated from whole snail tissues of all collected *Biomphalaria* using the BioSprint 96 workstation and BioSprint 96 DNA Blood Kit (QIAGEN Ltd., Manchester, UK) according to a previously outlined protocol that includes using double volume of lysis buffers and an overnight incubation lysis step [24, 29]. This magnetic bead-based DNA extraction protocol was chosen as it allows for rapid and high-throughput multiple-sample processing using a 96-well plate format.

#### *Biomphalaria* mitochondrial* cox*1 genotyping

Three randomly selected *Biomphalaria* collected from each malacological survey site across all surveys (*n* = 42 *Biomphalaria*) were genotyped, using PCR to amplify a 700-bp region of the *Biomphalaria* mitochondrial *cox*1 gene according to a standard protocol [9]. Details of all primer sequences, reaction mixes used and PCR conditions are described in Additional file 2: Tables S1A, S1B and S1C, respectively. All *Biomphalaria cox*1 PCRs included a positive control using *B. pfeifferi* DNA provided by the Schistosome and Snail Resource (SSR; an open access biomedical resource) [30] and a negative control using ddH_2_O in place of template DNA. Amplicons were visualised by running 4 µl of PCR products mixed with 1.5 µl of 5× loading buffer blue (Bioline Ltd., London, UK), stained with GelRed in a 1% agarose gel. The PCR products were then purified using the QIAquick PCR Purification Kit (QIAGEN Ltd.) according to the manufacturer’s instructions and Sanger sequenced in the forward direction using a dilution of the LCO_1490_FW forward primer. Sequence data were visualised, trimmed and edited as needed using Geneious Prime version 2023.01 (Biomatters Ltd., Aukland, New Zealand) before being identified using the Basic Local Alignment Search Tool (BLAST) algorithm within the National Center for Biotechnology Information (NCBI) database [31].

#### Phylogenetic and diversity analysis

##### Templeton Crandall and Sing haplotype network

To assess *B. pfeifferi cox*1 diversity, we performed a *cox*1 haplotype analysis using all generated *B. pfeifferi cox*1 sequence data as well as *cox*1 sequence data generated using eight *B. pfeifferi* recently collected in Chikwawa District, Malawi (approx. 250 km south of Mangochi District along the southern-flowing Shire River) (Nkolokosa et al*.,* under review), 13 East African *B. pfeifferi cox*1 sequences and five West African *B. pfeifferi cox*1 sequences (Additional file 3: Table S1). All *cox*1 data were aligned using the multiple alignment with he fast Fourier transform (MAFFT) algorithm within Geneious Prime (default MAFFT parameter settings). The MAFFT alignment was then visualised, trimmed (to ensure uniform ends across all sequences), examined and edited as needed before being exported from Geneious Prime in Nexus file format and imported into PopART version 1.7 [32]. Within PopART, a Templeton Crandall and Sing (TCS) haplotype network [33] was generated to allow examination of haplotype group structuring.

##### Maximum likelihood phylogenetic tree

To further assess *B. pfeifferi cox*1 diversity and lineages, we then aligned the MAFFT alignment to a *Biomphalaria glabrata cox*1 outgroup sequence (laboratory-bred 1742 strain, originating in Brazil [34]), as well as to a *Biophalaria sudanica cox*1 sequence (originating in Uganda, Lake Albert), both downloaded from the GenBank repository (Accession numbers: NC005439 and OQ849931, respectively). The alignment was visualised, trimmed, examined and edited as described above before being exported from Geneious Prime in Nexus file format and imported into MEGA11 [35]. Within MEGA11, a bootstrapped maximum likelihood (ML) phylogenetic tree was constructed using a Hasegawa-Kishino-Yano (HKY85) evolutionary model with 10,000 iterations after having calculated the most appropriate evolutionary model for phylogenetic analysis of these data.

### Molecular characterisation of *Schistosoma* cercariae

Individual *Schistosoma* cercariae were characterised using a multi-locus approach targeting both the mitochondrial (maternally inherited) *cox*1 and nuclear (inherited equally from both parents) ITS ribosomal RNA (rRNA) regions [36].

#### DNA extraction: FTA preserved cercariae

DNA was isolated from up to 20 individual Whatman FTA card-preserved *Schistosoma* cercariae, per shedding *Biomphalaria *snail, according to a standard protocol [37].

#### *Schistosoma* mitochondrial* cox*1 and nuclear ITS genotyping

A 956-bp region of the *Schistosoma* mitochondrial *cox*1 gene was amplified by PCR using a standard protocol [38]. Details of all primer sequences, reaction mixes used and PCR conditions are described in Additional file 2: Tables S2A, S2B and S2C, respectively. *Cox*1 PCRs included a positive control using *S. mansoni* genomic DNA (gDNA) collectively isolated from three *S. mansoni* adult worms provided by the SSR and a negative control using ddH_2_O in place of template DNA. Amplicons were visualised by running 4 µl of PCR products mixed with 1.5 µl of 5× loading buffer blue (Bioline Ltd.) stained with GelRed in a 1% agarose gel. PCR products were purified as described above and Sanger sequenced in the reverse direction using a dilution of the Schisto_3′ reverse primer. Sequence data were visualised, trimmed and edited as needed using Geneious Prime version 2023.01 (Biomatters Ltd.) before being identified using the BLAST algorithm within the NCBI database [31].

PCR then used to amplify the complete *Schistosoma* nuclear ITS rRNA region (approx. 1005 bp) using a standard protocol [38]. Details of all primer sequences, reaction mixes used, and PCR conditions are described in Additional file 2: Tables S3A, S3B and S3C, respectively. The ITS PCRs included one positive and one negative control as with *cox*1 PCRs. Amplicons were visualised as described above. PCR products were purified as described above and Sanger sequenced in the reverse direction using a dilution of the ETTS1 reverse primer. Sequence data were visualised, trimmed, edited and identified as with *cox*1 PCRs.

#### *Biomphalaria* molecular xenomonitoring

A recently developed high-throughput *S. mansoni*-specific molecular xenomonitoring PCR assay was used to detect either patent, but non-shedding, *S. mansoni* infections, or prepatent *S. mansoni* infections within *Biomphalaria* [24]. This assay can also be used to detect *Biomphalaria* infections with other trematode species, including *S. rodhaini.*

#### Molecular xenomonitoring PCRs

Molecular xenomonitoring was performed using DNA isolated from all *Biomphalaria* snails collected across all surveys. Details of all primer sequences, reaction mixes used and PCR conditions are described in Additional file 2: Tables S4A, S4B and S4C, respectively. Molecular xenomonitoring PCRs were carried out in batches of 92 samples in a 96-well PCR plate format. The PCR assays included one positive control using DNA) isolated from a non-infected *B. pfeifferi* provided by the SSR, one positive control using *S. mansoni* gDNA collectively isolated from three *S. mansoni* adult worms provided by the SSR, one positive control using *S. rodhaini* gDNA collectively isolated from three *S. rodhaini* adult worms provided by the SSR and one negative control using ddH_2_O in place of template DNA. PCR amplicons were visualised by running 7.5 µl PCR product mixed with 2 µl of 5× loading buffer blue (Bioline Ltd.) stained with GelRed in a 2% agarose gel.

Samples that amplified only the *Biomphalaria* ITS locus (considered an internal DNA extraction and PCR reaction control) were considered to be negative for Trematoda infection of any sort, including *S. mansoni*. Samples that successfully amplified all three target loci were considered positive for *S. mansoni* infection, as well as potentially for infection of other species of Trematoda. Samples that amplified only the *Biomphalaria* and Trematoda ITS loci were considered to be positive for non-*S. mansoni* Trematoda infection. Samples that failed to amplify the *Biomphalaria* ITS locus (regardless of Trematoda ITS and *S. mansoni* NADH dehydrogenase subunit 5 (ND5) amplification outcome), were considered to represent a failed PCR assay and the assay was repeated. Samples that failed to amplify this locus during the repeat screen were considered to represent a failed DNA extraction and were omitted from any further analysis.

#### Confirmatory* S. mansoni* ND5 genotyping

All samples that successfully amplified all three target loci were subjected to a secondary singleplex PCR to amplify only the *S. mansoni* ND5 locus for Sanger sequencing [24]. Amplicons were visualised in the same manner as for the multiplex molecular xenomonitoring PCRs. *Schistosoma mansoni* ND5 PCR products were purified as described above and Sanger sequenced in the forward direction using a dilution of the ND52 forward primer. Sequence data were visualised, trimmed, edited and identified as described above.

##### Templeton Crandall and Sing haplotype network

To assess *S. mansoni* ND5 diversity, we performed a haplotype analysis using all generated *S. mansoni* ND5 sequence data. To do this, a MAFFT alignment was performed using sequence data within Geneious Prime (default MAFFT parameter settings). The MAFFT alignment was then visualised, examined and edited as described above. The alignment was then exported from Geneious Prime in Nexus file format and imported into PopART version 1.7. Within PopART, a TCS haplotype network was generated to allow examination of haplotype group structuring.

#### Conformation of* Biomphalaria *infections with other Trematoda species

To identify Trematoda species other than *S. mansoni* infecting *Biomphalaria*, we subjected all samples that successfully amplified only *Biomphalaria* and Trematoda ITS loci to a secondary singleplex PCR, again to amplify only the *Biomphalaria* ITS and Trematoda ITS loci. To do this, the molecular xenomonitoring PCR was repeated, but with replacement of the *S. mansoni* ND5 forward and reverse primers with ddH_2_O. Amplicons were visualised in the same manner as for the multiplex molecular xenomonitoring PCRs and the approximately 1005-bp Trematoda ITS gel band was excised using a fresh scalpel. Excised gel bands were purified using the QIAquick Gel purification kit (Qiagen Ltd.) according to manufacturer’s instructions and were then purified as described above prior to Sanger sequencing in the forward direction using a dilution of the ETTS2 forward primer. Sequence data were visualised, trimmed and edited as needed using Geneious Prime version 2023.01 (Biomatters, Ltd.) before being identified using the BLAST algorithm within the NCBI database [31].

## Results

### Malacological surveillance and cercarial shedding of *Biomphalaria*

**Fig. 2:**
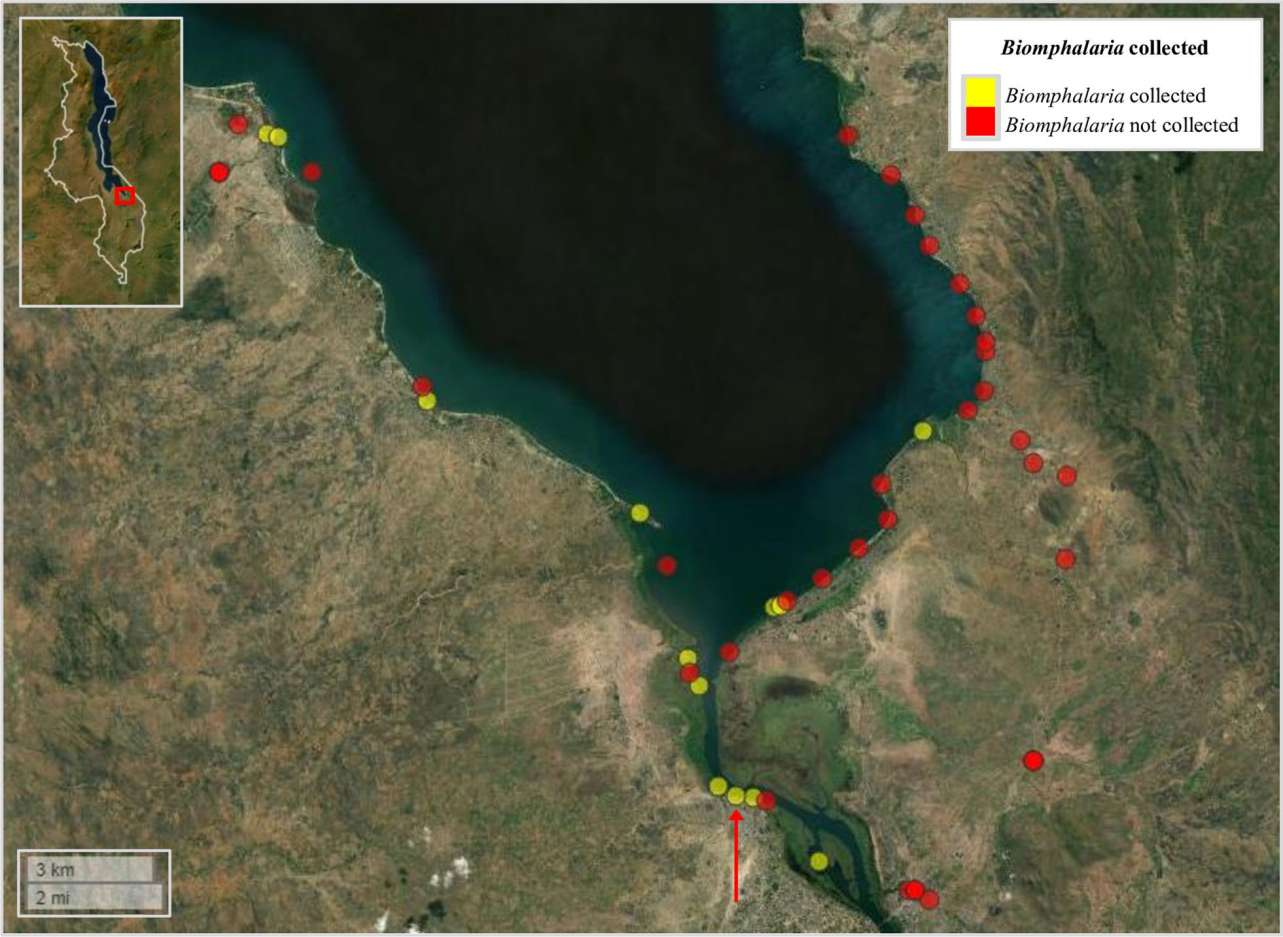
Overview of the 41 sites surveyed during malacological surveys 1, 2 and 3, Lake Malawi, Mangochi District, Malawi. Yellow circles denote sites where *Biomphalaria* were found during at least one of all three surveys. Red circles denote sites where *Biomphalaria* snails were not found during any of the three surveys (Additional file 1: Table S1). Only one *Biomphalaria* was found to be actively shedding *Schistosoma* cercariae (later identified as *S. mansoni*) during cercarial shedding analyses (during survey 3) at survey site 27; denoted by red arrow. Malawi’s country border can be seen within the figure inset (upper left corner). Inset: study area is highlighted by red box. Figure was generated using the ‘mapview’ package version 2.10.0 [39] within R Studio version 2021.09.0, build 351 (Posit, Boston, MA, USA) [40]

A total of 589 *Biomphalaria* were collected across all three surveys (survey 1: *n* = 285; survey 2: *n* = 107; survey 3: *n* = 197). Among all 41 sites surveyed, *Biomphalaria* were collected from 13 individual sites (31.7%) during at least one survey (Fig. 2; Additional file 1: Table S1), with all sites being either shallow temporal pools close to the lake’s edge or shallow waters along the shores of the lake. More sites were surveyed along the lake’s eastern shoreline owing to greater obstruction by dense vegetation on the lake’s western shoreline. The cercarial shedding analyses revealed that only one single *Biomphalaria* (0.17%), at survey site 27, was actively shedding *Schistosoma* cercariae (during survey 3) (Fig. 2; Additional file 1: Table S2). A total of 81 *Schistosoma* cercariae shed from this one snail were captured and preserved on a Whatman FTA card for subsequent molecular analysis.

### Molecular characterisation of *Biomphalaria*

**Fig. 3:**
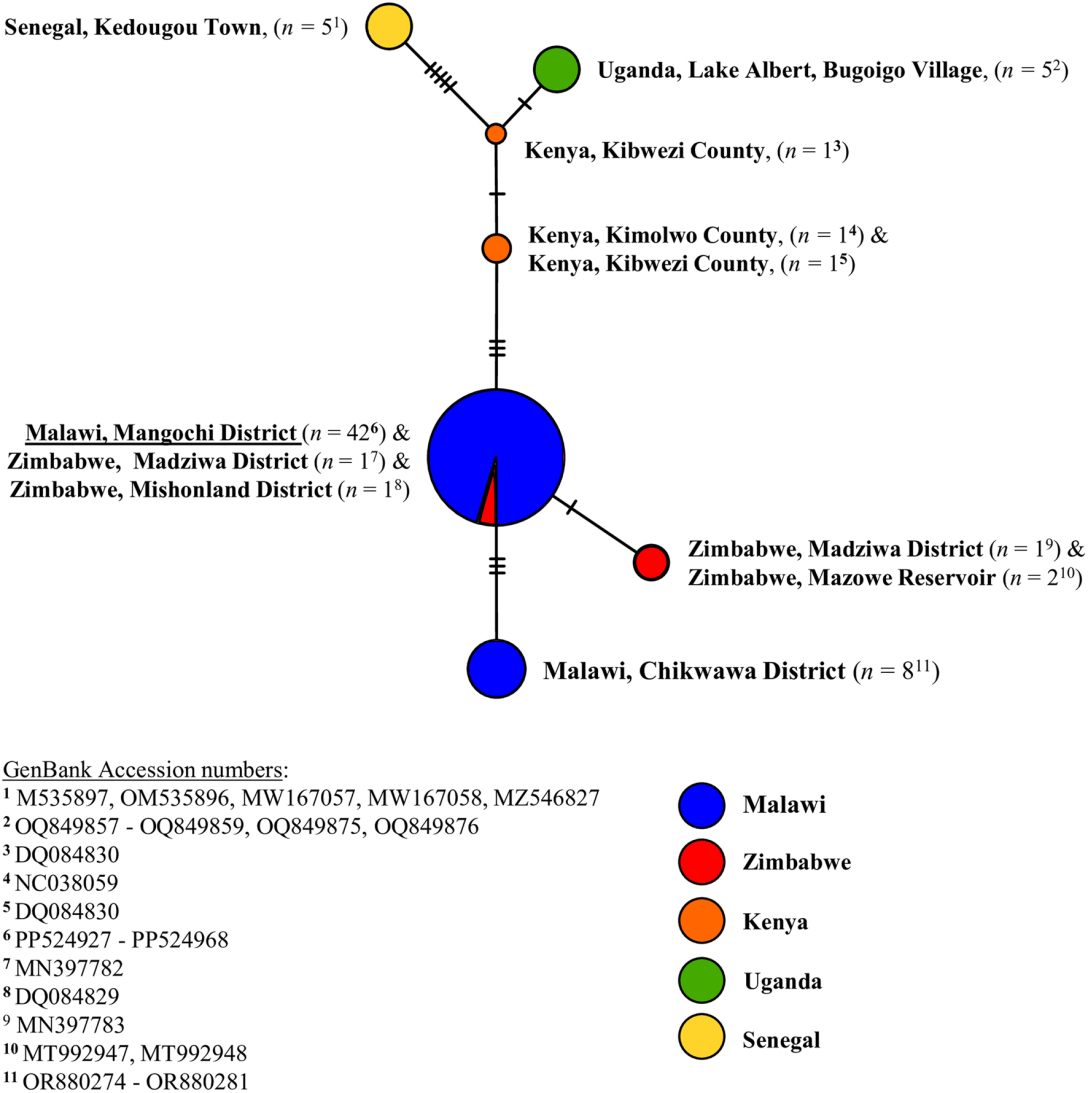
*Biomphalaria pfeifferi cox*1 TCS haplotype network. Each node (circle) represents a unique haplotype, with the size of the node proportional to the frequency of each haplotype. Each differently coloured node represents haplotype(s) from a different country, with blue denoting those haplotypes identified in Malawi; red, identified in Zimbabwe; orange, identified in Kenya; green, identified in Uganda; and yellow, identified in Senegal. The Mangochi District haplotype (current study) is underlined. Hatched lines denote the number of single nucleotide polymorphisms between nodes.* cox*1, Cytochrome oxidase subunit 1 gene; TCS, Templeton Crandall and Sing

**Fig. 4:**
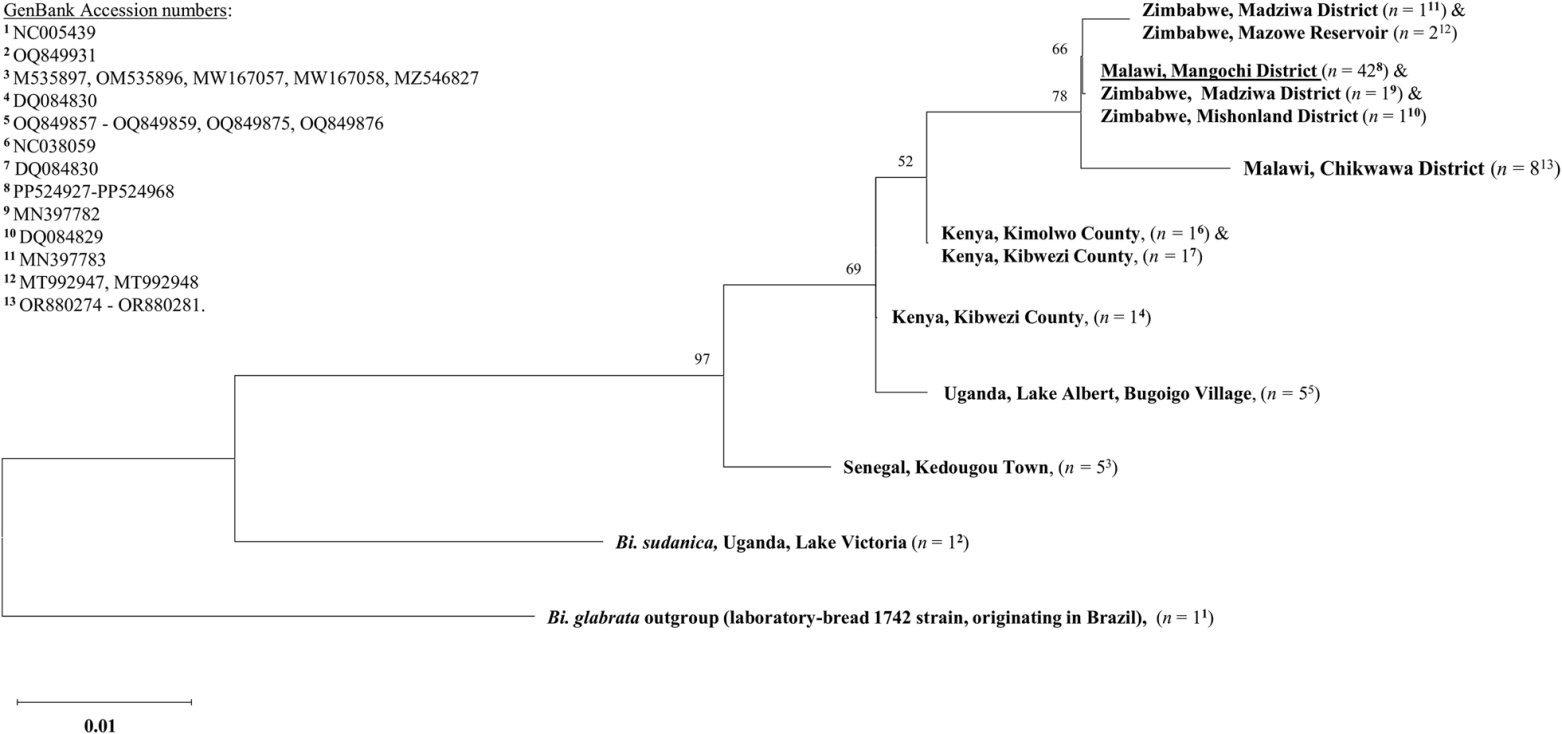
*Biomphalaria pfeifferi cox*1 maximum likelihood phylogenetic tree. Identical sequences were collapsed. Bootstrap values can be seen at branch nodes. The scale bar for branch length indicates the mean number of nucleotide substitutions per base site. A monophyletic clade was formed that included the Mangochi District *B. pfeifferi cox*1 haplotype (current study), all five Zimbabwe *B. pfeifferi cox*1 haplotypes and the Malawi, Chikwawa District *B. pfeifferi cox*1 haplotype. * cox*1, Cytochrome oxidase subunit 1 gene

DNA was successfully extracted from all collected *Biomphalaria* specimens. Of these, 42 were identified as *B. pfeifferi* though *cox*1 analysis. All 42 *B. pfeifferi cox*1 forward sequences were uploaded to the GenBank repository, (Accession numbers: PP524927-PP524968). The *B. pfeifferi cox*1 TCS haplotype network is shown in Fig. 3 and the *B. pfeifferi cox*1 ML phylogenetic tree is shown in Fig. 4.

No diversity was found between any Mangochi District *B. pfeifferi cox*1 sequence data (current study) with all samples representing a single haplotype. This haplotype was also identified in two *B. pfeifferi* from Zimbabwe (Madziwa District and Mishonland District) and was most closely related to a single *B. pfeifferi cox*1 haplotype found in three *B. pfeifferi* again from Zimbabwe (Madziwa District and Mazowe Reservoir). In addition, no diversity was found between any Malawi, Chikwawa District *B. pfeifferi cox*1 sequence data, and this haplotype was not found in any other *B. pfeifferi* analysed. Three single nucleotide polymorphisms (SNPs) were found to be common between the Mangochi District *B. pfeifferi cox*1 haplotype (current study) and the Malawi, Chikwawa District *B. pfeifferi cox*1 haplotype. Overall, very little *cox*1 diversity was observed across all *B. pfeifferi cox*1 data. However, different *B. pfeifferi cox*1 haplotypes were found from each analysed country (Senegal, Uganda, Kenya, Zimbabwe, and Malawi). Clustering was observed between haplotypes found in Zimbabwe and haplotypes found in Malawi, whereas the greatest degree of divergence was observed between haplotypes found in Senegal (West Africa) and haplotypes found in Malawi. This structuring was also observed within the ML phylogenetic tree, which formed a monophyletic clade that included the Mangochi District *B. pfeifferi cox*1 haplotype, all five Zimbabwe *B. pfeifferi cox*1 haplotypes and the Malawi, Chikwawa District *B. pfeifferi cox*1 haplotype.

### Molecular characterisation of *Schistosoma* cercariae

Five *Schistosoma* cercariae shed from the single shedding *Biomphalaria* were successfully genotyped to species level. All five were identified as *S. mansoni* across both *cox*1 and ITS loci. No genetic variation was observed between *cox*1 sequence data or between ITS sequence data. All five *S. mansoni cox*1 sequences and all five *S. mansoni* ITS sequences were uploaded to the GenBank repository (Accession numbers: PP529587-PP529591 and PP510205-PP510209, respectively).

### *Biomphalaria* molecular xenomonitoring

All 589 *Biomphalaria* snails were screened for *S. mansoni* and other Trematoda species infections using molecular xenomonitoring. A total of 14 (2.4%) samples failed to amplify the *Biomphalaria* internal control ITS locus, and so the amplifications were repeated using the same protocol, with all 14 successfully amplifying the *Biomphalaria* ITS locus during the repeat molecular xenomonitoring PCR. All three target loci were amplified when using DNA extracted from the one *B. pfeifferi* snail shedding *S. mansoni* cercariae, as well when using DNA isolated from 19 additional non-shedding *Biomphalaria*.

**Fig. 5:**
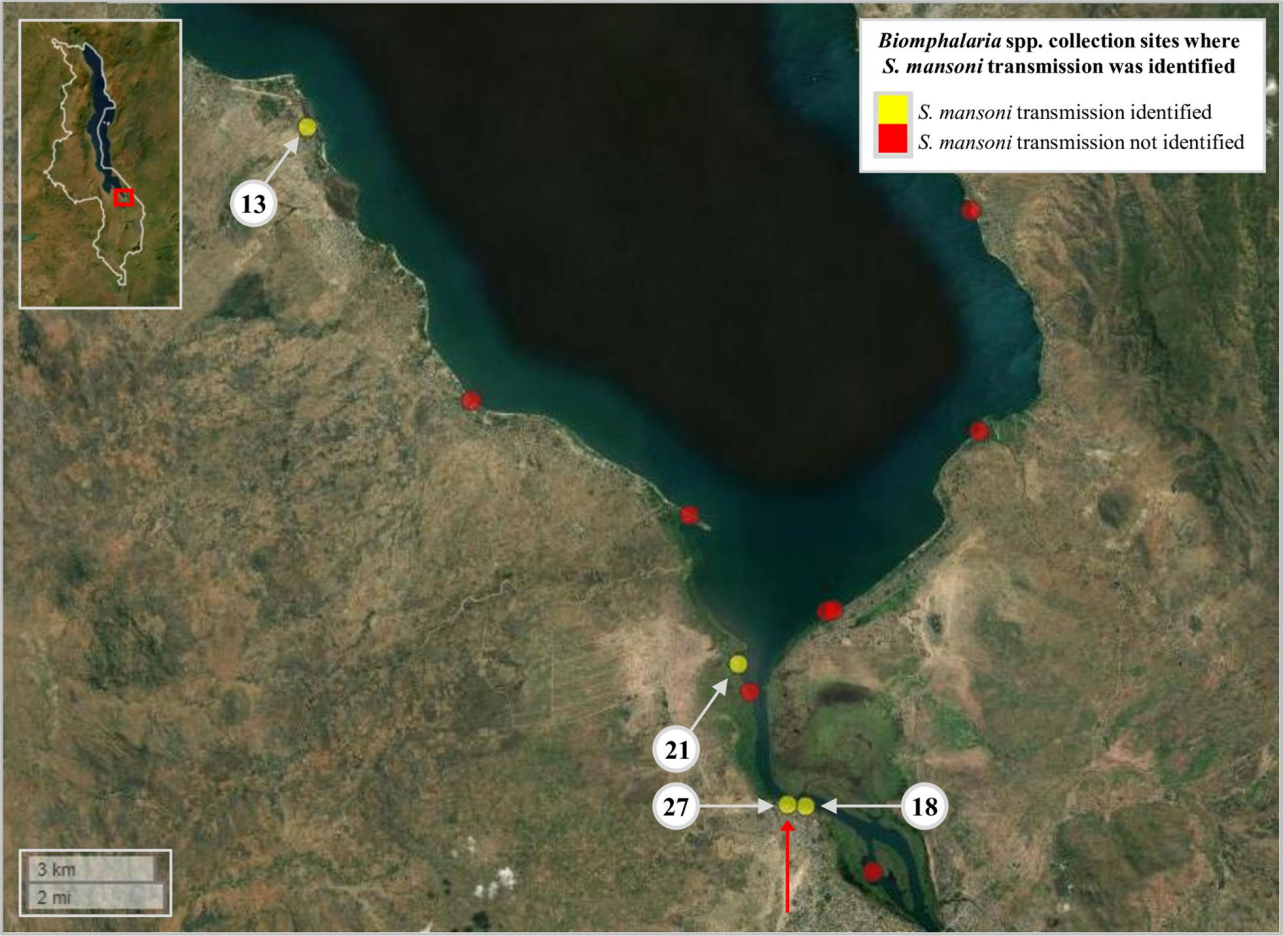
Malacological surveillance sites where *Biomphalaria* were collected and where *Schistosoma mansoni* transmission was or was not identified using molecular xenomonitoring. Site IDs are outlined in white circles (see Additional file 1: Tables S1, S2). The cercarial shedding analyses revealed that only one *Biomphalaria* (during survey 3) was actively shedding *Schistosoma* cercariae, which was later identified as *S. mansoni*, at survey site 27 (denoted with the red arrow). Malawi’s country border can be seen within figure inset (upper left corner). Within the inset, the study area is highlighted by a red box. Figure was generated using the ‘mapview’ package version 2.10.0 [39] within R Studio version 2021.09.0, build 351 (Posit, Boston, MA, USA) [40]

The *S. mansoni* ND5 locus was successfully amplified during the secondary *S. mansoni* ND5 singleplex PCR in all 20 *Biomphalaria* samples that had amplified all three target loci during the initial molecular xenomonitoring PCR. All 20 ND5 amplicons were confirmed as *S. mansoni* through ND5 analysis, confirming infection with *S. mansoni*. The prevalence of *S. mansoni* infection in these 589 *Biomphalaria* was therefore increased from 0.17% based on cercarial shedding to 3.4% based on molecular xenomonitoring. *Biomphalaria* snails infected with *S. mansoni* were identified at four of the 41 (9.6%) malacological survey sites (Fig. 5). At site 13, three of 175 collected *Biomphalaria* (1.7%) were infected with *S. mansoni*; at site 18, two of 66 collected *Biomphalaria* (3%) were infected with *S. mansoni*; and at site 27, five of 151 collected *Biomphalaria* (4%) were infected with *S. mansoni*. At site 21, however, nine of 80 *Biomphalaria* (11.25%) were infected with *S. mansoni*, the highest prevalence of *Biomphalaria* infections across all malacological surveillance sites. The number of malacological surveillance sites where intestinal schistosomiasis transmission was identified was therefore increased from just one using cercarial shedding to four using molecular xenomonitoring.

**Fig. 6:**
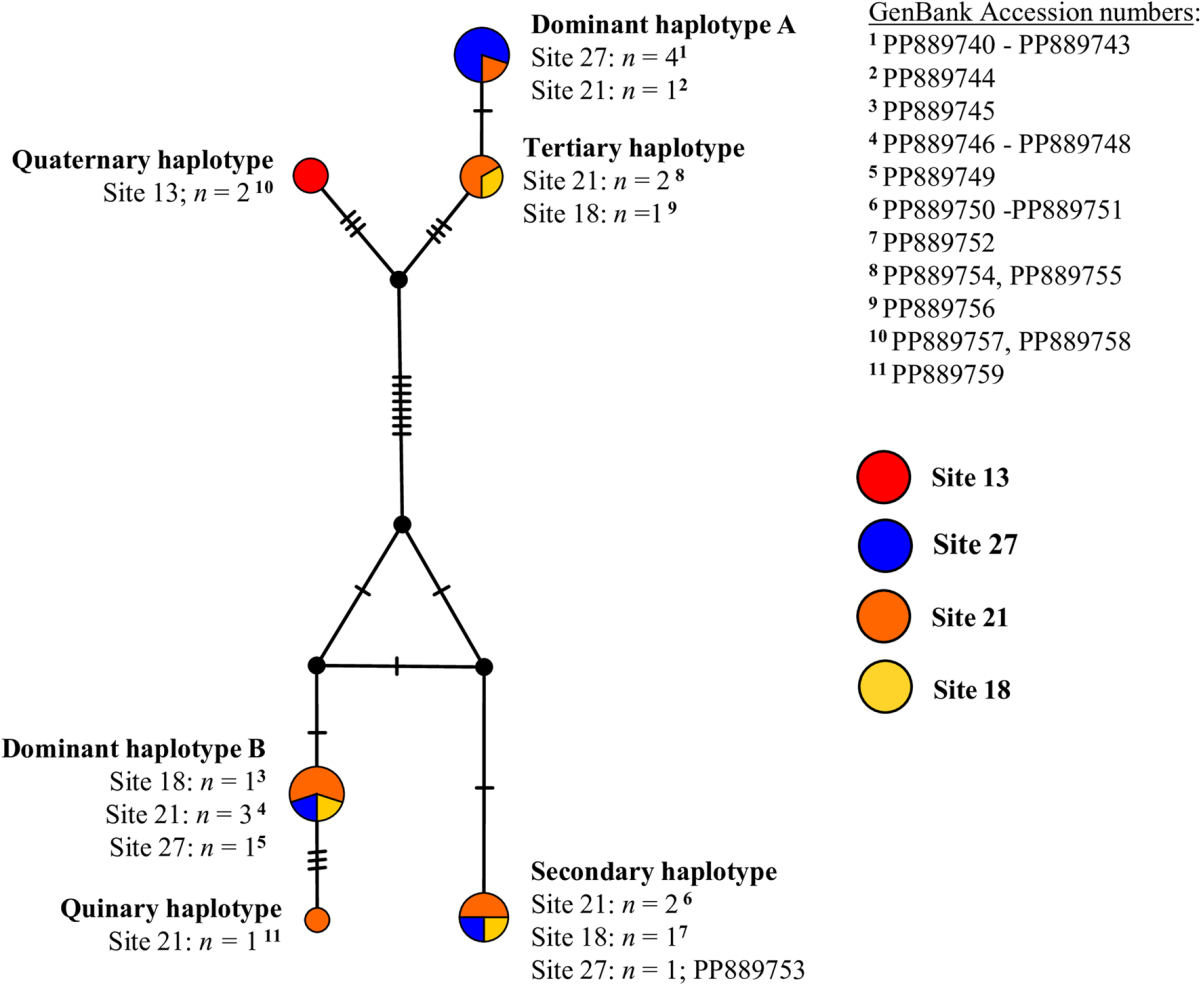
*Schistosoma mansoni* ND5 TCS haplotype network. Each node (circle) represents a unique haplotype, with the size of the node proportional to the frequency of each haplotype. Different node colours represent a specific haplotype(s) identified at that site, with red denoting the haplotype identified at site 13; blue denoting haplotypes identified at site 27; orange denoting haplotypes identified at site 21; and yellow denoting haplotypes identified at site 18. Hatched lines denote the number of SNPs between nodes. ND5, NADH dehydrogenase subunit 5; SNP, single nucleotide polymorphism; TCS, Templeton Crandall and Sing

The *S. mansoni* ND5 TCS haplotype network is shown in Fig. 6. Two distinct clusters were formed, each comprising three unique haplotypes. One unique haplotype consisting of two *S. mansoni* ND5 sequences was identified at site 13; no other haplotypes were found at this site. One unique haplotype comprising one *S. mansoni* ND5 sequence was identified at site 21. The remaining four haplotypes were present at more than one single site, with two being present at three sites (sites 18, 21 and 27). All 20 *S. mansoni* ND5 sequences were uploaded to the GenBank repository (Accession numbers: PP889740-PP889759).

Only the *Biomphalaria* ITS and Trematoda ITS loci were amplified in 12 *Biomphalaria* DNA samples, indicating infection with non-*S. mansoni* trematodes. The Trematoda ITS gel band was successfully excised and genotyped in all 12 of these samples. Of these 12 samples, six were identified as *Uvulifer* spp., which were all present at site 13, and the remaining six were identified as *Petasiger* spp., which were present at sites 18, 20, 21 and 27 (Additional file 1: Table S2), based on ITS analysis. No genetic variation was found between any ITS sequence data for *Uvulifer* spp. or between any ITS sequence data for *Petasiger* spp. All six *Uvulifer* spp. ITS sequences and all six *Petasiger* spp. ITS sequences were uploaded to the GenBank repository (Accession numbers: PP510464-PP510469 and PP510476-PP501481, respectively). An example agarose gel image of the high-throughput molecular xenomonitoring PCR assay [24] is shown in Additional file 4: Figure S1. Additional data generated during Biomphalaria molecular xenomonitoring can be found in Additional file 5: Dataset S1, Biomphalaria molecular xenomonitoring data.

## Discussion

In the present study, we carried out extensive malacological surveillance along the southern shoreline of Lake Malawi, Malawi to measure the distribution and diversity of *Biomphalaria* freshwater snail intermediate hosts of *S. mansoni*. We then used a range of molecular approaches to genotype a subset of collected *Biomphalaria* snails to species level and to screen all collected *Biomphalaria* for infection with *S. mansoni* to identify intestinal schistosomiasis transmission sites in this area. The overall aim was to gain a more thorough understanding of *Biomphalaria*-associated transmission of intestinal schistosomiasis in Mangochi District five years post-outbreak to better inform and strengthen future disease control efforts.

*Biomphalaria* were collected at multiple surveillance sites, and all genotyped specimens were identified as *B. pfefferi,* which is the most widely distributed and commonly implicated intermediate host of *S. mansoni* across sub-Saharan Africa [25]. No *cox*1 variation was found between all *B. pfeifferi* genotyped in Mangochi District, suggesting a potential single and recent colonisation event of *B. pfeifferi* in this area. According to *cox*1 analysis, all *B. pfeifferi* genotyped here were most closely related to *B. pfeifferi* collected in Zimbabwe, with two Zimbabwean isolates from different locations sharing identical *cox*1 sequence data to Mangochi District *B. pfeifferi*; it is therefore possible that these *B. pfeifferi* identified in Mangochi District originated from Zimbabwe, as has been suggested previously [22]. Interestingly, sufficient genetic variation was found between the unique Malawi, Chikwawa District haplotype and the Malawi, Mangochi District haplotype to suggest that the Chikwawa District *B. pfeifferi* population may have in fact invaded from populations outside of Malawi, rather than migrated south along the Shire River from Mangochi District and diverged, as might have been initially assumed. Further analysis to confirm this possibility, however, is needed, particularly as the *cox*1 phylogenetic tree generated here suggests that all Malawian *B. pfeifferi* (including those from Mangochi and Chikwawa Districts) and all Zimbabwean *B. pfeifferi* share a common ancestor. It is worth noting that no other species of *Biomphalaria*, such as *B. sudanica* or *B. choanomphala*, was identified, although limited surveillance in deep water (> 1 m) took place, which is the preferred microhabitat of *B. choanomphala* [25].

This recent introduction of *Biomphalaria* into Mangochi District can likely be, in part, attributed to environmental changes caused by ongoing climate change [41, 42]. In recent years, Mangochi District—and Malawi more broadly—have been severely impacted by atypical and extreme weather conditions, such as tropical cyclones and remarkably heavy rainfall with subsequent flooding, particularly in low-altitude areas such as the upper Shire River margins [43]. Whilst flooding can transport and introduce *Biomphalaria* into new habitats from surrounding areas, such extreme weather events can also dramatically alter freshwater environments more generally, generating more favourable *Biomphalaria* habitats [41, 42]. As an example, temporal freshwater pools, which would typically evaporate during dry seasons, may instead remain as permanent waterbodies in which *B. pfeifferi* can propagate. Such waterbodies may therefore quickly become active sites of intestinal schistosomiasis transmission. As different *Biomphalaria* species, which can differ in their ability to transmit *S. mansoni*, have different habitat preferences, a clear understanding of how climate change can alter the environment with respect to current and potential *Biomphalaria* habitats is needed to better understand ongoing and future transmission of intestinal schistosomiasis. Molecular approaches to identify *Biomphalaria* species, such as those used here, will be crucial in these investigations.

As found previously in this area [22], just one (0.17%) of all 589 collected *Biomphalaria* snails was found to be actively shedding *S. mansoni* cercariae. Molecular xenomonitoring, however, revealed that a total of 20 (3.4%) *Biomphalaria* were infected with *S. mansoni*, 12 of which were collected from malacological surveillance sites that were not deemed intestinal schistosomiasis transmission sites based on cercarial shedding analysis. Consequently, the prevalence of *Biomphalaria* infection with *S. mansoni* increased from 0.17% to 3.4%, and the number of identified intestinal schistosomiasis transmission sites increased from one to four, based on the results of molecular xenomonitoring. Interestingly, at one such site (site 21; see Additional file 1: Table S1), not only was a relatively high number of *Biomphalaria* collected (*n* = 80), but a high proportion (11.25%) of *Biomphalaria* were found to be infected with *S. mansoni* using molecular xenomonitoring despite none of these actively shedding *S. mansoni* cercariae during the cercarial shedding analysis. Furthermore, of note, this site is a short distance (approx. 1.5 km) from Samama School, at which 55% of school-aged children in attendance were found to be infected with *S. mansoni* during recent parasitological surveillance in November 2021 [Archer et al., under review].

In addition, molecular xenomonitoring identified six unique *S. mansoni* mitochondrial ND5 haplotypes, which clustered into two distinct groups. A similar degree of mitochondrial DNA genetic diversity was found amongst *S. mansoni* miracidia *cox*1 haplotypes (infecting school-aged children) also recently identified in this area (Archer et al. under review). Furthermore, during these previous *cox*1 analyses, two genetically distinct *S. mansoni cox*1 linage groups (II and IV) [44, 45] were identified within the miracidia population, which may be reflected here in these ND5 haplotypes. Interestingly, it should also be noted that just one unique *S. mansoni* ND5 haplotype was identified at site 13, which is far removed (approx. 12–15 km) from the remaining three surveillance sites where *S. mansoni* transmission was found (all of which are within approx. 3 km of each other), suggesting a focal population of *S. mansoni* at site 13 that is genetically distinct from *S. mansoni* populations further south along the lake’s shoreline. At the remaining three intestinal schistosomiasis transmission sites (sites 18, 21 and 27), multiple *S. mansoni* haplotypes were identified and found to be present at multiple sites, suggesting mixing of *S. mansoni* populations in this area, likely by human hosts visiting multiple different lake water contact points but also potentially through movement of *Biomphalaria* between surveyed sites.

Infection with non-*Schistosoma* trematodes was also detected in 12 *Biomphalaria* collected at multiple malacological surveillance sites. Six of these were *Petasiger* spp., which were present at sites 18, 20, 21 and 27. *Petasiger* spp. trematodes have three hosts, namely, multiple genera of freshwater snails including *Biomphalaria*; freshwater fish and tadpoles; and piscivorous birds such as cormorants [46]. The remaining six were *Uvulifer* spp., which were all present at site 13. *Uvulifer* spp. trematodes also have three hosts: multiple genera of freshwater snails, again including *Biomphalaria;* freshwater sunfish; and piscivorous birds such as kingfisher [47]. Whilst there appears to be no available studies investigating whether established *Uvulifer* spp. or *Petasiger* spp. infections within *Biomphalaria* impact *S. mansoni* development and transmission, other trematode species have been found to influence *S. mansoni* development within *Biomphalaria* [48], and this may also be the case for these infecting trematodes. This possibility, however, requires further investigation to clarify.

### Study limitations and future work

Given the limited diversity found across *B. pfeifferi cox*1 haplotypes analysed in this study, a more thorough analysis of additional *B. pfeifferi* DNA loci, such as the mitochondrial 16S or nuclear ITS regions, or even whole mitochondrial genome analysis, would likely provide further insights into Mangochi District *B. pfeifferi* phylogenies, population structuring and origins [10]. In addition, whilst molecular xenomonitoring can be used to detect *Schistosoma* infections in freshwater snail hosts that have been missed by cercarial shedding examination, this approach does have limitations. PCR-based molecular approaches, such as end-point PCR used here or real-time/quantitative PCR, are expensive and require specialised laboratory personnel. The continued development of more portable and easy-to-use nucleic acid amplification technologies that could be performed in schistosomiasis-endemic areas, such as loop-mediated isothermal amplification (LAMP) and recombinase polymerase/aided amplification (RPA/RAA), for molecular xenomonitoring purposes is therefore encouraged here [49, 50]. The continued development of DNA extraction technologies capable of isolating DNA from freshwater snail tissues in resource-poor settings is also encouraged [50].

## Conclusions

Through extensive malacological surveillance and the use of molecular approaches, we confirm that *Biomphalaria* are now present at many locations along the southern shoreline of Lake Malawi, Malawi. We also confirm that many of these locations are active intestinal schistosomiasis transmission sites. When compared to the results of previous malacological surveys in this area, it would appear that *B. pfeifferi* populations are rapidly expanding, not only across this southern shoreline of Lake Malawi, but also further south of Malawi in Chikwawa District. As such, the revision of ongoing schistosomiasis control programmes in Mangochi District, such as the implementation of bi-annual MDA using praziquantel, significantly improving access to adequate water and sanitation hygiene (WASH) infrastructure, freshwater snail population control and continued delivery of schistosomiasis education and health programmes to promote behaviours that limit the risk of contracting and transmitting schistosomiasis, is therefore encouraged, in line with WHO recommendations. Our study also highlights the importance of molecular approaches to investigate *Biomphalaria* populations and monitor *Biomphalaria*-associated intestinal schistosomiasis transmission in endemic areas. As such, the continued development and use of such approaches, in particular the development and use of molecular xenomonitoring assays that can be carried out in resource-poor schistosomiasis-endemic settings, is also encouraged. These will be especially needed in low-endemicity areas and will aid in improving disease control efforts, significantly reducing disease-related morbidities experienced by those afflicted by intestinal schistosomiasis.

## Supplementary Information

Additional file 1: Table S1. *Biomphalaria *collected during three malacological surveys carried out along the southern shoreline of Lake Malawi, Malawi. Table S2. Cercarial shedding and molecular xenomonitoring of collected Biomphalaria.

Additional file 2: Table S1A. Primer sequences used to detect and amplify a 700-bp fragment of the *Biomphalaria *spp. mitochondrial cytochrome oxidase subunit 1 (*cox*1) gene. Table S1B. Reaction mix used to carry out endpoint PCR to detect and amplify a 700-bp fragment of the *Biomphalaria *spp. mitochondrial cytochrome oxidase subunit 1 (*cox*1) gene. Table S1C. PCR conditions used to carry out endpoint PCR to detect and amplify a 700-bp fragment of the *Biomphalaria *spp. mitochondrial cytochrome oxidase subunit 1 (*cox*1) gene. Table S2A. Primer and probe sequences used to amplify a 956-bp region of the *Schistosoma *spp. mitochondrial cytochrome oxidase subunit 1 (*cox*1) gene. Table S2B. Reaction mix used to carry out end-point targeting a 956-bp region of the *Schistosoma *spp. mitochondrial cytochrome oxidase subunit 1 (*cox*1) gene. Table S2C. PCR conditions used to carry out end-point targeting a 956 bp region of the *Schistosoma *spp. mitochondrial cytochrome oxidase subunit 1 (*cox*1) gene. Table S3A. Primer and probe sequences used to amplify the complete* Schistosoma *spp. nuclear internal transcribed spacer region (inclusive of both ITS regions 1 + 2 and the nuclear 5.8S region). Table S3B. Reaction mix used to carry out end-point PCR targeting the complete* Schistosoma *spp. nuclear internal transcribed spacer region (inclusive of both ITS regions 1 + 2 and the nuclear 5.8S region). Table S3C. PCR conditions used to carry out end-point PCR targeting the complete* Schistosoma *spp. nuclear internal transcribed spacer region (inclusive of both ITS regions 1 + 2 and the nuclear 5.8S region). Table S4A. Primer sequences used to detect and amplify the complete *Biomphalaria *spp. and* Schistosoma *spp. nuclear internal transcribed spacer regions (~ 1250 bp and ~ 1005bp in length, respectively; inclusive of both ITS regions 1 + 2 and the nuclear 5.8S region). Table S4B. Molecular xenomonitoring PCR reaction mix used to detect and amplify the complete *Biomphalaria *spp. ITS region, the complete *Trematoda* ITS region and a partial region of the *S. mansoni *ND5 gene. Table S4C. Molecular xenomonitoring PCR cycling conditions used to detect and amplify the complete *Biomphalaria *spp. ITS region, the complete *Trematoda*. ITS region and a partial region of the *S. mansoni *ND5 gene.

Additional file 3: Table S1. *B. pfeifferi cox*1 data and GenBank accession numbers used for *cox*1 haplotype analysis.

Additional file 4: Figure S1. Example agarose gel image of the high-throughput molecular xenomonitoring PCR assay

Additional file 5: Dataset S1. *Biomphalaria *molecular xenomonitoring data.

## Data Availability

All data generated during malacological collections,* Biomphalaria *cercarial shedding analyses and* Biomphalaria* molecular xenomonitoring can be found in Additional file 1: Table S1. All data on* Biomphalaria* collected during three malacological surveys carried out along the southern shoreline of Lake Malawi, Malawi can be found in Additional file 1: Table S2, cercarial shedding and molecular xenomonitoring of collected* Biomphalaria*. An example agarose gel image of the high-throughput molecular xenomonitoring PCR assay [24] is shown in Additional file 4: Figure S1. Additional data generated during* Biomphalaria* molecular xenomonitoring can be found in Additional file 5: Dataset S1,* Biomphalaria* molecular xenomonitoring data. All Sanger sequence data generated was uploaded to the GenBank repository as described (*B. pfeifferi cox*1 sequences: GenBank accession numbers PP524927-PP524968;* S. mansoni cox*1 sequences: GenBank accession numbers PP529587-PP529591;* S. mansoni* ITS sequences: GenBank accession numbers PP510205-PP510209;* S. mansoni* ND5 sequences: GenBank accession numbers PP889740–PP889759;* Uvulifer* spp. ITS sequences: GenBank accession numbers PP510464-PP510469;* Petasiger* spp. ITS sequences: GenBank accession numbers PP510476-PP501481).

## Abbreviations

BLAST: Basic Local Alignment Search Tool
*Cox*1: Cytochrome oxidase subunit 1 gene
ITS: Internal transcribed spacer
LAMP: Loop-mediated isothermal amplification
MAFFT: Multiple alignment with fast Fourier transform
ML: Maximum likelihood
NCBI: National Center for Biotechnology Information
ND5: NADH dehydrogenase subunit 5
NTD: Neglected tropical disease
RPA/RAA: Recombinase polymerase/aided amplification
SNP: Single nucleotide polymorphism
SSR: Schistosome and Snail Resource
TCS network: Templeton Crandall and Sing haplotype network
WASH: Water and sanitation hygiene
