# Supplementary material for: Population genetics and molecular xenomonitoring of Biomphalaria freshwater snails along the southern shoreline of Lake Malawi, Malawi
Source: Parasit Vectors. 2024 Dec 18;17:521. doi: 10.1186/s13071-024-06546-5 (PMC11657217; doi:10.1186/s13071-024-06546-5)
Supplement: Supplementary file 1 — Additional file 1: Table S1. Biomphalaria collected during three malacological surveys carried out along the southern shoreline of Lake Malawi, Malawi. Table S2. Cercarial shedding and molecular xenomonitoring of collected Biomphalaria. [file 13071_2024_6546_MOESM1_ESM.docx]

Population genetics and molecular xenomonitoring of *Biomphalaria* freshwater snails along the southern shoreline of Lake Malawi, Mangochi District, Malawi

**Additional file 1: Malacological surveillance and molecular xenomonitoring data**

**Table S1.** *Biomphalaria* collected during three malacological surveys carried out along the southern shoreline of Lake Malawi, Malawi.

| **Malacological survey name** | **Survey date** | **Latitude** | **Longitude** | **Site ID** | **Site ID (previous)** | **Number of *Biomphalaria* collected** | ***Biomphalaria* species (confirmed through *cox*1 genotyping of three specimens)** |
| --- | --- | --- | --- | --- | --- | --- | --- |
| HUGS* pilot | November 2021 | -14.31373 | 35.14174 | S13 | HUGS16/ Mangochi 1 | 101 | *Bi. pfeifferi* |
| HUGS* pilot | November 2021 | -14.39261 | 35.2218 | S14 | HUGS17 | 3 | *Bi. pfeifferi* |
| HUGS* pilot | November 2021 | -14.4648 | 35.26014 | S16 | HUGS20 | 7 | *Bi. pfeifferi* |
| HUGS* pilot | November 2021 | -14.45161 | 35.24633 | S18 | HUGS22 | 62 | *Bi. pfeifferi* |
| HUGS* pilot | November 2021 | -14.44942 | 35.23875 | S19 | HUGS23 | 50 | *Bi. pfeifferi* |
| HUGS* pilot | November 2021 | -14.42852 | 35.23436 | S20 | HUGS24 | 71 | *Bi. pfeifferi* |
| HUGS* pilot | November 2021 | -14.41223 | 35.25069 | S24 | HUGS28 | 4 | *Bi. pfeifferi* |
| HUGS* baseline | July 2022 | -14.31414 | 35.14407 | S13 | HUGS16/ Mangochi 1 | 6 | *Bi. pfeifferi* |
| HUGS* baseline | July 2022 | -14.42255 | 35.23224 | S21 | HUGS5/ Mangochi 4 | 80 | *Bi. pfeifferi* |
| HUGS* baseline | July 2022 | -14.41184 | 35.252107 | S29 | HUGS.B | 18 | *Bi. pfeifferi* |
| HUGS* baseline | July 2022 | -14.37558 | 35.282599 | S34 | HUGS.G12A | 1 | *Bi. pfeifferi* |
| HUGS* baseline | July 2022 | -14.33057 | 35.280838 | S39 | HUGS.K | 1 | *Bi. pfeifferi* |
| HUGS* E4 | October/ November 2022 | -14.31414 | 35.14407 | S13 | HUGS16/ Mangochi 1 | 50 | *Bi. pfeifferi* |
| HUGS* E4 | October/ November 2022 | -14.36915 | 35.17623 | S1 | HUGS0/ Mangochi 3 | 1 | *Bi. pfeifferi* |
| HUGS* E4 | October/ November 2022 | -14.42255 | 35.23224 | S21 | HUGS25/ Mangochi 4 | 2 | *Bi. pfeifferi* |
| HUGS* E4 | October/ November 2022 | -14.45145 | 35.24238 | S27 | Mangochi 5/ River Bend | 144 | *Bi. pfeifferi* |

**Where:*

HUGS: Hybridisations in UroGenital Schistosomiasis study

**Table S2.** Cercarial shedding and molecular xenomonitoring of collected *Biomphalaria.*

| **Malacological survey name** | **Site ID** | **Number of *Biomphalaria* collected** | **Number of *Biomphalaria* shedding *S. mansoni* cercariae (confirmed through *cox*1 and ITS genotyping)** | ***Biomphalaria* infection with *S. mansoni* confirmed through molecular xenomonitoring** | ***Biomphalaria* infection with other Trematoda species confirmed through molecular xenomonitoring** | **Trematoda species (confirmed through ITS genotyping)** |
| --- | --- | --- | --- | --- | --- | --- |
| HUGS* pilot | S13 | 101 | 0 | 2 (2%) | 4 (4%) | *Uvulifer* sp. |
| HUGS* pilot | S18 | 62 | 0 | 2 (3.2%) | 1 (1.6%) | *Petasiger* sp. |
| HUGS* pilot | S20 | 71 | 0 | 0 | 1 (1.2%) | *Petasiger* sp. |
| HUGS* baseline | S21 | 81 | 0 | 9 (11%) | 3 (3.7%) | *Petasiger* sp |
| HUGS* E4 | S13 | 50 | 0 | 1 (2%) | 2 (4%) | *Uvulifer* sp. |
| HUGS* E4 | S27 | 144 | 1 (1.8%) | 6 (4.2%) | 0 | NA |

**Where:*

HUGS: *Hybridisations in UroGenital Schistosomiasis* study
