# Supplementary material for: Population genetics and molecular xenomonitoring of Biomphalaria freshwater snails along the southern shoreline of Lake Malawi, Malawi
Source: Parasit Vectors. 2024 Dec 18;17:521. doi: 10.1186/s13071-024-06546-5 (PMC11657217; doi:10.1186/s13071-024-06546-5)
Supplement: Supplementary file 2 — Additional file 2: Table S1A. Primer sequences used to detect and amplify a 700-bp fragment of the Biomphalaria spp. mitochondrial cytochrome oxidase subunit 1 (cox1) gene. Table S1B. Reaction mix used to carry out endpoint PCR to detect and amplify a 700-bp fragment of the Biomphalaria spp. mitochondrial cytochrome oxidase subunit 1 (cox1) gene. Table S1C. PCR conditions used to carry out endpoint PCR to detect and amplify a 700-bp fragment of the Biomphalaria spp. mitochondrial cytochrome oxidase subunit 1 (cox1) gene. Table S2A. Primer and probe sequences used to amplify a 956-bp region of the Schistosoma spp. mitochondrial cytochrome oxidase subunit 1 (cox1) gene. Table S2B. Reaction mix used to carry out end-point targeting a 956-bp region of the Schistosoma spp. mitochondrial cytochrome oxidase subunit 1 (cox1) gene. Table S2C. PCR conditions used to carry out end-point targeting a 956 bp region of the Schistosoma spp. mitochondrial cytochrome oxidase subunit 1 (cox1) gene. Table S3A. Primer and probe sequences used to amplify the complete Schistosoma spp. nuclear internal transcribed spacer region (inclusive of both ITS regions 1 + 2 and the nuclear 5.8S region). Table S3B. Reaction mix used to carry out end-point PCR targeting the complete Schistosoma spp. nuclear internal transcribed spacer region (inclusive of both ITS regions 1 + 2 and the nuclear 5.8S region). Table S3C. PCR conditions used to carry out end-point PCR targeting the complete Schistosoma spp. nuclear internal transcribed spacer region (inclusive of both ITS regions 1 + 2 and the nuclear 5.8S region). Table S4A. Primer sequences used to detect and amplify the complete Biomphalaria spp. and Schistosoma spp. nuclear internal transcribed spacer regions (~ 1250 bp and ~ 1005bp in length, respectively; inclusive of both ITS regions 1 + 2 and the nuclear 5.8S region). Table S4B. Molecular xenomonitoring PCR reaction mix used to detect and amplify the complete Biomphalaria spp. ITS region, the comp [file 13071_2024_6546_MOESM2_ESM.docx]

Population genetics and molecular xenomonitoring of *Biomphalaria* freshwater snails along the southern shoreline of Lake Malawi, Mangochi District, Malawi

**Additional file 2:** **Molecular methods**

Biomphalaria *spp. mitochondrial* cox*1 PCR and genotyping*

**Table S1A:** Primer sequences used to detect and amplify a 700-bp fragment of the *Biomphalaria* spp. mitochondrial cytochrome oxidase subunit 1 (*cox*1) gene.

| **Name** | **Target** | **Oligonucleotide sequence (5’ – 3’)** | **Reference** |
| --- | --- | --- | --- |
| **LCO_1490_FW*** | *Biomphalaria* spp. *cox*1 locus | GGTCAACAAATCATAAAGATATTGG | (1) |
| **HCO_2198_RV**^†^ |  | TAAACTTCAGGGTGACCAAAAAATCA |  |

**Forward primer*

^†^*Reverse primer*

**Table S1B:** Reaction mix used to carry out endpoint PCR to detect and amplify a 700-bp fragment of the *Biomphalaria* spp. mitochondrial cytochrome oxidase subunit 1 (*cox*1) gene.

|  | **Concentration** | **Volume per 1 sample (µl))** |
| --- | --- | --- |
| ddH_2_O | / | 22 |
|  |  |  |
| LCO_1490_FW* | 10 pmol | 1 |
| HCO_2198_RV^†^ | 10 pmol | 1 |
|  |  |  |
| Illustra PuReTaq ready-to-go PCR bead  [Sigma-Aldrich, USA] | / | **/** |
|  |  |  |
| DNA | / | 1*^‡^* |
|  |  | **25** |

**Forward primer*

^†^*Reverse primer*

*^‡^DNA template diluted 1:10 using molecular grade H_2_O*

**Table S1C:** PCR conditions used to carry out endpoint PCR to detect and amplify a 700-bp fragment of the *Biomphalaria* spp. mitochondrial cytochrome oxidase subunit 1 (*cox*1) gene.

| **PCR step** | **Time** | **Temp** | **Cycles** |
| --- | --- | --- | --- |
| Denaturation | 5 min | 95 ºC | / |
| Annealing | 30 sec | 95 ºC | 40 |
|  | 30 sec | 44 ºC |  |
|  | 1.30 min | 72 ºC |  |
| Extension | 10 min | 72 ºC | / |

*Mitochondrial* cox*1 genotyping:* Schistosoma *spp. cercariae*

**Table S2A:** Primer and probe sequences used to amplify a 956 bp region of the *Schistosoma* spp. mitochondrial cytochrome oxidase subunit 1 (*cox*1) gene.

| **Name** | **Target** | **Oligonucleotide sequence (5’ – 3’)** | **Reference** |
| --- | --- | --- | --- |
| **Schisto_5’*** | *Schistosoma* spp. *cox*1 locus | TCTTTRGATCATAAGCG | (2). |
| **Schisto_3’**^†^ |  | TAATGCATMGGAAAAAAACA |  |

**Forward primer*

^†^*Reverse primer*

**Table S2B:** Reaction mix used to carry out end-point targeting a 956 bp region of the *Schistosoma* spp. mitochondrial cytochrome oxidase subunit 1 (*cox*1) gene.

|  | **Concentration** | **Volume per 1 sample (µl)** |
| --- | --- | --- |
| ddH_2_O | / | 20 |
|  |  |  |
| Schisto_5’* | 10 pmol | 1 |
| Schisto_3’^†^ | 10 pmol | 1 |
|  |  |  |
| Illustra PuReTaq ready-to-go PCR bead  [Sigma-Aldrich, USA] | / | **/** |
|  |  |  |
| DNA | / | 3 |
|  |  | **25** |

**Forward primer*

^†^*Reverse primer*

**Table S2C:** PCR conditions used to carry out end-point targeting a 956 bp region of the *Schistosoma* spp. mitochondrial cytochrome oxidase subunit 1 (*cox*1) gene.

| **PCR step** | **Time** | **Temp** | **Cycles** |
| --- | --- | --- | --- |
| Denaturation | 5 min | 95 ºC | / |
| Annealing | 30 sec | 95 ºC | 40 |
|  | 30 sec | 40 ºC |  |
|  | 1.30 min | 72 ºC |  |
| Extension | 10 min | 72 ºC | / |

*Nuclear ITS genotyping:* Schistosoma *spp. cercariae*

**Table S3A:** Primer and probe sequences used to amplify the complete *Schistosoma* spp. nuclear internal transcribed spacer region (inclusive of both ITS regions 1 + 2 and the nuclear 5.8S region).

| **Name** | **Target** | **Oligonucleotide sequence (5’ – 3’)** | **Reference** |
| --- | --- | --- | --- |
| **ETTS2*** | *Schistosoma* spp. ITS locus | TAACAAGGTTTCCGTAGGTGA | (3). |
| **ETTS1**^†^ |  | TGCTTAAGTTCAGCGGG |  |

**Forward primer*

^†^*Reverse primer*

**Table S3B:** Reaction mix used to carry out end-point PCR targeting the complete *Schistosoma* spp. nuclear internal transcribed spacer region (inclusive of both ITS regions 1 + 2 and the nuclear 5.8S region).

|  | **Concentration** | **Volume per 1 sample (µl)** |
| --- | --- | --- |
| ddH_2_O | / | 20 |
|  |  |  |
| ETTS2* | 10 pmol | 1 |
| ETTS1^†^ | 10 pmol | 1 |
|  |  |  |
| Illustra PuReTaq ready-to-go PCR bead  [Sigma-Aldrich, USA] | / | **/** |
|  |  |  |
| DNA | / | **3** |
|  |  | **25** |

**Forward primer*

^†^*Reverse primer*

**Table S3C:** PCR conditions used to carry out end-point PCR targeting the complete *Schistosoma* spp. nuclear internal transcribed spacer region (inclusive of both ITS regions 1 + 2 and the nuclear 5.8S region).

| **PCR step** | **Time** | **Temp** | **Cycles** |
| --- | --- | --- | --- |
| Denaturation | 5 min | 95 ºC | / |
| Annealing | 30 sec | 95 ºC | 40 |
|  | 30 sec | 58 ºC |  |
|  | 1.30 min | 72 ºC |  |
| Extension | 10 min | 72 ºC | / |

*Molecular xenomonitoring of collected* Biomphalaria *spp. specimens*

**Table S4A.** Primer sequences used to detect and amplify the complete *Biomphalaria* spp. and *Schistosoma* spp. nuclear internal transcribed spacer regions (~1250-bp and ~1005-bp in length, respectively; inclusive of both ITS regions 1 + 2 and the nuclear 5.8S region).

| **Name** | **Target** | **Oligonucleotide sequence (5’ – 3’)** | **Reference** |
| --- | --- | --- | --- |
| ETTS2* | *Biomphalaria* ITS (~1250-bp) & *Schistosoma* ITS (~1005-bp) | TGCTTAAGTTCAGCGGG | (5) |
| ETTS1^†^ |  | TAACAAGGTTTCCGTAGGTGA |  |
| ND52* | *S. mansoni* ND5 (305-bp) | ATTAGAGGCAATGCGTGCTC | (6) |
| ND52^†^ |  | ATTGAACCAACCCCAAATCA |  |

**Forward primer*

^†^*Reverse primer*

**Table S4B:** Molecular xenomonitoring PCR reaction mix used to detect and amplify the complete *Biomphalaria* spp. ITS region, the complete *Trematoda*. ITS region, and a partial region of the *S. mansoni* ND5 gene.

|  | **Concentration** | **Volume per 1 sample (µl)** |
| --- | --- | --- |
| ddH_2_O | / | 19 |
|  |  |  |
| ETTS2* | 10 pmol | 1 |
| ETTS1^†^ | 10 pmol | 1 |
| ND52* | 10 pmol | 1 |
| ND52^†^ | 10 pmol | 1 |
|  |  |  |
| Illustra PuReTaq ready-to-go PCR bead [Sigma-Aldrich, USA] | / | **/** |
|  |  |  |
| DNA | / | 2 |
|  |  | **25** |

**Forward primer*

^†^*Reverse primer*

**Table S4C:** Molecular xenomonitoring PCR cycling conditions used to detect and amplify the complete *Biomphalaria* spp. ITS region, the complete *Trematoda*. ITS region, and a partial region of the *S. mansoni* ND5 gene.

| **PCR step** | **Time** | **Temp °C** | **Cycles** |
| --- | --- | --- | --- |
| Denaturation | 5 minutes | 95 | / |
| Annealing | 30 seconds | 95 | 40 |
|  | 30 seconds | 60 |  |
|  | 90 seconds | 72 |  |
| Extension | 10 minutes | 72 | / |

**References**

1. Folmer O, Black M, Hoeh W, Lutz R, Vrijenhoen R. DNA primers for amplification of mitochondrial cytochrome c oxidase subunit I from diverse metazoan invertebrates. *Molecular Marine Biology and Biotechnology*. 1994. 3(5):294-299.

2. Lockyer AE, Olson PD, Østergaard P, Rollinson D, Johnston DA, Attwood SW, *et al*. The phylogeny of the Schistosomatidae based on three genes with emphasis on the interrelationships of *Schistosoma* Weinland, 1858. *Parasitology*. 2003. 126(3):203–24.

3. Kane RA, Ridgers IL, Johnston DA, Rollinson D. Repetitive sequences within the first internal transcribed spacer of ribosomal DNA in schistosomes contain a Chi-like site. *Molecular and Biochemical Parasitology.* 1996. 75(2):265–9.

4. Archer J, Yeo SM, Gadd G, Pennance T, Cunningham LJ, Juhàsz A, et al. Development, validation, and pilot application of a high throughput molecular xenomonitoring assay to detect *Schistosoma mansoni* and other trematode species within *Biomphalaria* freshwater snail hosts. *Current Research in Parasitology & Vector-Borne Diseases*. 2024. 5:100174.

5. Kane RA, Rollinson D. Repetitive sequences in the ribosomal DNA internal transcribed spacer of *Schistosoma haematobium*, *Schistosoma intercalatum* and *Schistosoma mattheei*. *Molecular and Biochemical Parasitology*. 1994. 63(1):153–6.

6. Lu L, Zhang SM, Mutuku MW, Mkoji GM, Loker ES. Relative compatibility of *Schistosoma mansoni* with *Biomphalaria sudanica* and *B. pfeifferi* from Kenya as assessed by PCR amplification of the *S. mansoni* ND5 gene in conjunction with traditional methods. *Parasites and Vectors*. 2016. 9(1):1–13.
