# Supplementary material for: Population genetics and molecular xenomonitoring of Biomphalaria freshwater snails along the southern shoreline of Lake Malawi, Malawi
Source: Parasit Vectors. 2024 Dec 18;17:521. doi: 10.1186/s13071-024-06546-5 (PMC11657217; doi:10.1186/s13071-024-06546-5)
Supplement: Supplementary file 3 — Additional file 3: Table S1. B. pfeifferi cox1 data and GenBank accession numbers used for cox1 haplotype analysis. [file 13071_2024_6546_MOESM3_ESM.docx]

Population genetics and molecular xenomonitoring of *Biomphalaria* freshwater snails along the southern shoreline of Lake Malawi, Mangochi District, Malawi

**Additional file 3: *Biomphalaria pfeifferi cox*1 reference data and GenBank accession numbers used for phylogenetic analyses.**

**Table S1.** *B. pfeifferi cox*1 data and GenBank accession numbers used for *cox*1 haplotype analysis.

| **Country** | **Location** | **Number of *cox*1 isolates** | **GenBank accession numbers** |
| --- | --- | --- | --- |
| Uganda | Lake Albert Bugoigo Village | 5 | OQ849857  OQ849858  OQ849859  OQ849875  OQ849876 |
|  | Lake Victoria | 0 (non-available) | NA |
|  | Lake Kyoga | 0 (non-available) | NA |
|  | Lake Edward | 0 (non-available) | NA |
| Kenya | Lake Victoria Homa Bay | 1 | OL423116 |
|  | Kimolwo County | 1 | NC038059 |
|  | Kibwezi County | 1 | DQ084830 |
| Tanzania | NA | 0 (non-available) | NA |
| Rwanda | NA | 0 (non-available) | NA |
| Burundi | NA | 0 (non-available) | NA |
| Democratic Republic of the Congo | NA | 0 (non-available) | NA |
| Zambia | NA | 0 (non-available) | NA |
| Mozambique | NA | 0 (non-available) | NA |
| Zimbabwe | Mashonaland Central Province | 1 | DQ084829 |
|  | Madziwa District | 2 | MN397782  MN397783 |
|  | Mazowe Reservoir | 2 | MT992947  MT992948 |
| Cameroon | NA | 0 (non-available) | NA |
| Côte d’lvoire | NA | 0 (non-available) | NA |
| Senegal | Kedougou Town | 2 | M535897  OM535896 |
|  | Unknown | 3 | MW167057  MW167058  MZ546827 |
| The Gambia | NA | 0 (non-available) | NA |
