# Supplementary figures and images for: Population genetics and molecular xenomonitoring of Biomphalaria freshwater snails along the southern shoreline of Lake Malawi, Malawi
Source: Parasit Vectors. 2024 Dec 18;17:521. doi: 10.1186/s13071-024-06546-5 (PMC11657217; doi:10.1186/s13071-024-06546-5)

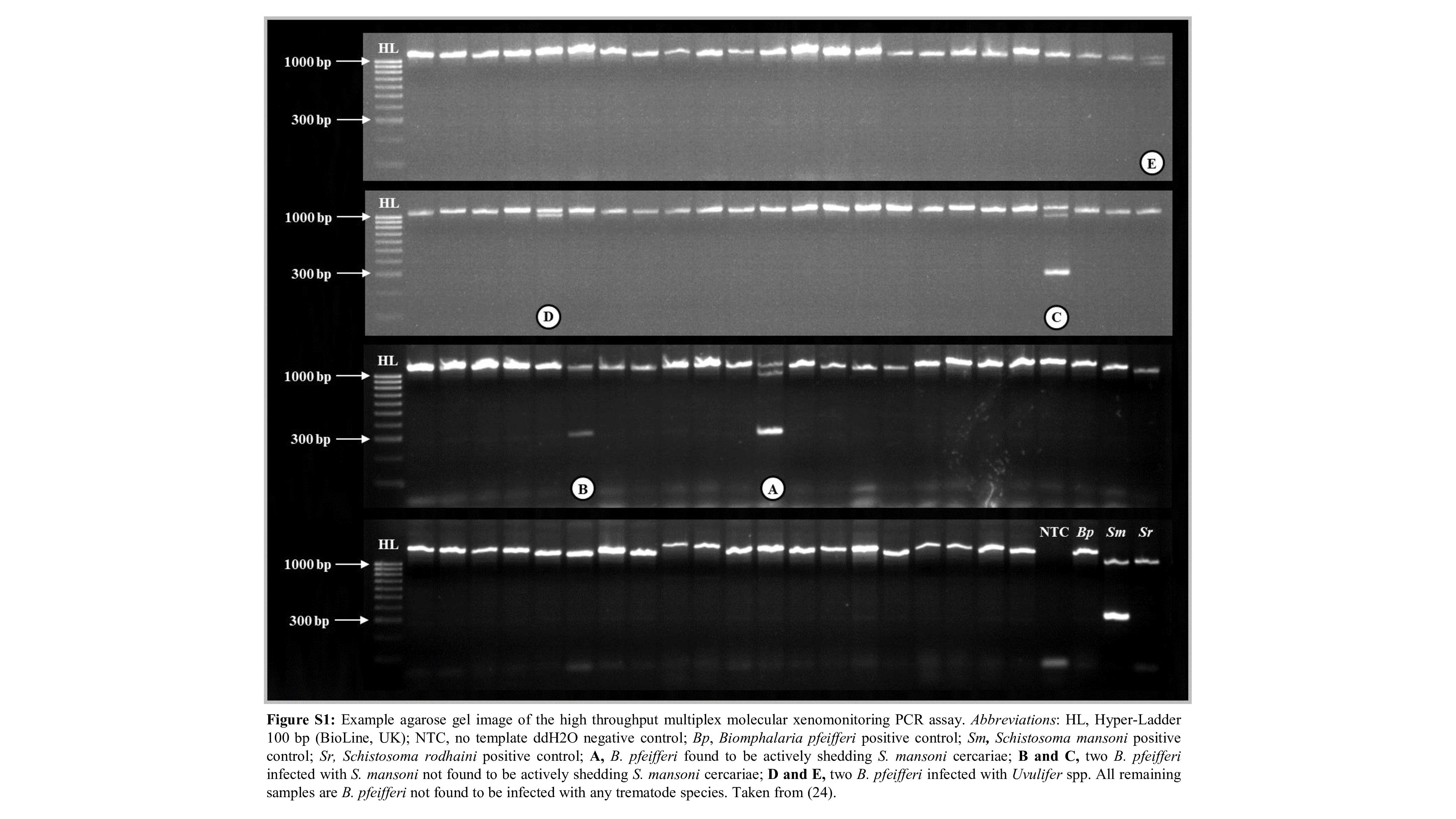

Supplement: Supplementary file 4 — Additional file 4: Figure S1. Example agarose gel image of the high-throughput molecular xenomonitoring PCR assay [file 13071_2024_6546_MOESM4_ESM.tiff]
